# Treatment of metal (loid) contaminated solutions using iron-peat as sorbent: is landfilling a suitable management option for the spent sorbent?

**DOI:** 10.1007/s11356-019-05379-5

**Published:** 2019-05-23

**Authors:** Alfreda Kasiuliene, Ivan Carabante, Prosun Bhattacharya, Jurate Kumpiene

**Affiliations:** 1 Department of Civil, Environmental and Natural Resources Engineering, Lulea University of Technology, SE-97187 Lulea, Sweden; 2 Department of Sustainable Development, Environmental Science and Engineering, Royal Institute of Technology, Teknikringen 76, SE-100 44 Stockholm, Sweden

**Keywords:** Arsenic, Metals, Trace elements, Sorption, Landfill leachate, Low redox

## Abstract

This study firstly aimed to investigate the potential of simultaneous metal (loid) removal from metal (oid) solution through adsorption on iron-peat, where the sorbent was made from peat and Fe by-products. Up-flow columns filled with the prepared sorbent were used to treat water contaminated with As, Cu, Cr, and Zn. Peat effectively adsorbed Cr, Cu, and Zn, whereas approximately 50% of inlet As was detected in the eluent. Iron-sand was effective only for adsorbing As, but Cr, Cu, and Zn were poorly adsorbed. Only iron-peat showed the simultaneous removal of all tested metal (loid)s. Metal (loid) leaching from the spent sorbent at reducing conditions as means to assess the behaviour of the spent sorbent if landfilled was also evaluated. For this purpose, a standardised batch leaching test and leaching experiment at reducing conditions were conducted using the spent sorbent. It was found that oxidising conditions, which prevailed during the standardised batch leaching test, could have led to an underestimation of redox-sensitive As leaching. Substantially higher amounts of As were leached out from the spent sorbents at reducing atmosphere compared with oxidising one. Furthermore, reducing environment caused As(V) to be reduced into the more-toxic As (III).

## Introduction

Arsenic (As) contaminates soil and groundwater in various areas throughout Sweden due to past industrial activities, such as glass works and wood impregnation, and other metals, such as chromium (Cr), copper (Cu), and zinc (Zn), occur as co-contaminants at varying concentrations in some of these sites (Bhattacharya et al. 2002; Lov et al. 2017; Hagner et al. 2018).

Arsenic is commonly removed from contaminated water by adsorption onto reactive media. Iron (Fe) oxides have an affinity for arsenates and are known to be effective and potentially inexpensive adsorbents for use in treating As-contaminated water (Carabante et al. 2014; Ahmad et al. 2017). To avoid clogging of filters (Mohan and Pittman 2007) and to overcome low hydraulic permeability (Theis et al. 1992), Fe-containing filters are commonly produced by coating Fe oxides onto a bulk material, such as sand (Devi et al. 2014; Wang et al. 2017; Callegari et al. 2018) or activated carbon (Yurum et al. 2014; Mondal and Garg 2017), where there are advantages of using activated carbon instead of sand because it also targets other contaminants.

Peat is among the numerous natural materials that has a metal adsorption capacity (Chaney and Hundemann 1979), and it can be coated with Fe oxides without employing an activation step (in contrast to many of the materials used to produce activated carbon). As shown by Kasiuliene et al. (2018), combining peat with Fe results in a versatile sorbent that can simultaneously target both cationic and anionic contaminants. This preparation provides the potential of utilising by-products and/or waste materials when manufacturing the adsorbent. Peat as waste can occur from the production of energy and electricity, or from agriculture, horticulture, and water filtration processes, and iron by-products can be obtained from the steel industry. In this respect, utilisation of by-products and waste materials is an environmentally sustainable practice that contributes to a circular economy.

Landfilling is often the preferred option for spent adsorbents that bear inorganic contaminants (Mohan and Pittman 2007). The most common method to determine landfill type for waste acceptance is the compliance batch leaching test when waste is exposed to a leachant for 24 h under aerobic conditions (Council Directive 1991/31/EC, Annex II). The purpose of the test is to simulate the leaching potential of the waste placed in landfill. However, landfills normally provide anaerobic conditions, unlike the aerobic conditions that are present in the standardised leaching test, and thus the standardised leaching test will not always adequately address the actual leaching potential of investigated waste.

In a low redox environment, arsenates (As(V)) can be reduced to arsenites (As (III)), which are more mobile and toxic (Corvin et al. 1999). Whereas, Cr (VI) can undergo very rapid changes to poorly soluble Cr (III) if redox potential drops (Hausladen and Fendorf 2017). Zinc, and especially Cu, usually remains insoluble as precipitates in a landfill environment (Jambeck et al. 2008). The solubility of metal (loid)s can change due to water infiltration and the influx of oxygen (O_2_) into the landfill site, and due to changes in biological activity (Bozkurt et al. 2000). This necessitates leachate treatment leading to a perpetual cycle. Thus, when evaluating metal (loid) releases from landfills over a long-time, abiotic factors such as redox potential need to be seriously considered.

This study firstly aimed to investigate the potential of simultaneous metal (loid) removal from solution through adsorption on iron-peat, where the sorbent was made from peat and Fe by-products. Up-flow columns filled with the prepared sorbent were used to treat water contaminated with As, Cu, Cr, and Zn. The second aim of this study was to evaluate metal (loid) leaching from the spent sorbent at reducing conditions, with the objective of assessing the behaviour of the spent sorbent if it were to be landfilled. For this purpose, a standardised batch leaching test and leaching experiment at reducing conditions were conducted.

## Material and methods

### Preparation of sorbents

Heat-treated peat powder was obtained from Geogen Produktion AB, Sweden. The company produces heat-treated peat granulates as an environmentally compatible oil-adsorption agent, and particles smaller than 2 mm are discarded during its production. Ferric-ferrous hydrosol provided by Rekin, Lithuania, was used to coat the peat. The hydrosol was a colloidal suspension of Fe (II) and Fe (III) hydrated compounds; it is produced from Fe waste during the process of electrolysis and acts as a binder for wastewater pollutants. The peat powder was then thoroughly mixed with the ferric-ferrous hydrosol at a ratio of 1*w*:1*w*, with a small amount of water added for homogenisation.

To verify that both peat and Fe are required for the simultaneous removal of cations and anions, in the following experiments, two control sorbents were used: i) sand, with a particle size of 0.5–1.0 mm, coated with ferric-ferrous hydrosol (1*w*:4*w*) and ii) uncoated peat. Hereinafter, coated peat powder is referred to as ‘iron-peat’, coated sand as ‘iron-sand’, and control peat powder as ‘peat’.

All sorbents were then dried at 35 °C for 24 h before being used as up-flow column fillers. Total solids (TS) and loss on ignition (LOI) were subsequently determined following standard procedures (ISO 11465: 1993); the specific surface area was determined according to the Brunauer–Emmett–Teller (BET) nitrogen (N_2_) adsorption isotherm technique (where N_2_ adsorption was conducted at 77 K) using an ASAP 2020 micropore analyser (Micrometrics).

### Column adsorption experiment

A known mass of dry peat (110 g), iron-peat (134 g), and iron-sand (546 g) sorbents was sealed into high-density polyethylene columns (45 mm × 200 mm) with a bed volume (BV) of 0.32 L. The inlet hose was connected to a container holding a metal (loid) solution, which is hereafter referred to as the ‘inlet solution’, and the outlet hose was connected to a sample collection bottle that was air-tight and dark to avoid Fe oxidation. The experiment was performed in triplicate. The flow rate was set at 1.2 mL min^−1^. The eluate was sampled daily, and electrical conductivity (EC) (CDM 10, Radiometer Copenhagen), pH (pH 340, WTW), and Eh (CDM 10, Radiometer Copenhagen) were measured immediately after sampling. Subsamples for metal (loid) analysis were filtered through 0.45-μm cellulose filters, acidified and analysed using inductively coupled plasma optical emission spectrometry (ICP-OES) (Optima 8300, Perkin-Elmer). The experiment was stopped after 60 days; the columns were then opened and the spent sorbents were laid out to dry at 35 °C for 24 h. The total metal (loid) concentrations in the sorbents before and after the filter experiment were determined with ICP-OES after wet digestion with *aqua regia* in a microwave (CEM Mars 5) at 190 °C.

The metal (loid) solution was prepared from analytical grade chemicals: NaH_2_AsO_4_ (Honeywell Riedel-de Haën AG, 99%), K_2_Cr_2_O_7_ (VWR International, 99.9%), CuCl_2_·2H_2_O (Merck, 99%), and ZnCl_2_ (Merck, 98%). Metal (loid) salts were dissolved into 0.1 M potassium nitrate (KNO_3_, VWR International, 100%) solution, and the solution pH was set to 5.0 using 0.1 M hydrochloric acid (HCl, Merck, 37%). Initial metal (loid) concentrations in the solution were determined using ICP-OES, and detection limits were 0.002 mg L^−1^ for As; 0.001 mg L^−1^ for Cr, Cu, and Zn; and 0.014 mg L^−1^ for Fe. The inlet solution contained 0.88 ± 0.07 mg L^−1^ of As and 4.1 ± 0.4 mg L^−1^of Cr, Cu, and Zn.

### Standardised leaching test

A batch leaching test at a liquid-to-solid (L/S) ratio = 10, following procedure described in the Council Decision 2003/33/EC, was performed with spent sorbents that were obtained after conducting the column adsorption experiment. Samples were leached with ultra-pure water for 24 h using an end-over-end rotator, then filtered and immediately analysed for pH, EC, and Eh. Metal (loid) concentrations were determined using ICP-OES. A total organic carbon analyser (TOC-L series, Shimadzu) was used to determine the dissolved carbon (DC) content of the leachates. The detection limits for dissolved organic carbon (DOC) and for dissolved inorganic carbon (DIC) were 4 μg L^−1^.

### Leaching test at reducing conditions

Spent sorbents and ultra-pure water at L/S = 10 were sealed in 1-L glass bottles, and each replicate contained material from a separate column filter. Air in the bottles was exchanged with a methane (CH_4_) and carbon dioxide (CO_2_) gas mixture (50:50), and the bottles were placed in a dark cabinet at a temperature of 30 ± 3 °C. For sampling, 5–6 mL of the liquid was taken through a long needle inserted into the bottles, and the liquid was then measured immediately for Eh, pH, and EC. Subsamples were filtered, acidified, and analysed using ICP-OES for metal (loid)s. Gasses in the bottles were supplemented with the CH_4_ and CO_2_ mixture after sampling the liquid. The gas composition (CO_2_, O_2_, N_2_, and CH_4_) was analysed at the end of the experiment using a gas chromatograph (Clarus 500) to verify the presence of reducing conditions. The detection limit was 100 ppm for all gases. At the end of the experiment, the DOC content was determined as described in the “Standardised leaching test” section. The experiment lasted 200 days.

### Landfill gas production

A biochemical methane potential (BMP) test was conducted in 100-mL gas-tight serum bottles. The inoculated medium contained ultra-pure water, 2 mL of nutrients (Cederroth Nutrient Bloom containing *N*_total_ 5.1 g, potassium (K) 4.3 g, calcium (Ca) 0.3 g, phosphorus (P) 1 g, sulphur (S) 0.4 g, magnesium (Mg) 0.4 g, Fe 35 mg, manganese (Mn) 20 mg, boron (B) 10 mg, Zn 3.0 mg, Cu 1.5 mg, and molybdenum (Mo) 0.4 mg; diluted at the ratio 1:10) per 1 g TS of the spent sorbent, and the inoculum (comprising sewage sludge after anaerobic digestion), which was added at a ratio of 3*w*:1*w* of the volatile solids (VS). Calculation of VS was based on LOI (loss on ignition) analysis. Spent sorbent samples corresponding to 3 g of TS were added into the bottles together with the medium, and the bottles were sealed and incubated at 30 °C. Control bottles contained only the inoculated medium. The volume of gas produced was measured regularly. Since iron-sand contained only a negligible fraction of VS (< 1%), it was not used in the BMP test because it was assumed that it would produce negligible amounts of landfill gases.

### Speciation analysis

The Cr speciation in column adsorption samples was determined after 5 and after 50 days of the experiment as well as in the inlet solution. Determination of total Cr and Cr (VI) was done by ion chromatography, and Cr (III) was calculated from measured values. The detection limit for the total Cr and Cr (VI) was 0.4 μg L^−1^.

The As speciation in leachate samples was determined after the standardised batch leaching test and after the low redox experiment final sampling (after 200 days). Samples were analysed using liquid chromatography-inductively coupled plasma mass spectrometry. The detection limit for As (III), As(V), and dimethylarsinic acid (DMA) was 0.1 μg L^−1^; and that for methylarsonic acid (MMA) was 0.2 μg L^−1^.

Samples were kept frozen in plastic bottles prior to analysis. Both Cr and As speciation was done at an accredited laboratory (ALS Scandinavia).

### Statistics

The data were processed using an analysis of variance (ANOVA) test with the software Minitab 18. A two-sample *t* test (*p* > 0.05) was applied to differentiate between sample means.

## Results

### Performance of column filters

Three sorbents (peat, iron-peat, and iron-sand) were tested in up-flow columns for adsorption of contaminants from a solution. The metal (loid) contents of freshly prepared sorbents obtained prior to the adsorption experiment are presented in Table 1. Peat-based sorbents contained 21–22 mg As kg^−1^. Arsenic is an intrinsic element in peat, with concentrations ranging from 1 ng L^−1^ (Stepanova et al. 2015) up to 1800 mg kg^−1^ (Langner et al. 2013). Peat contained low levels of Cr and Cu, which increased in iron-peat and iron-sand due to the ferric-ferrous hydrosol coating, where Cu and Cr may occur as impurities. Zinc was present both in peat and in sand. Uncoated peat contained approximately 10 g Fe kg^−1^, but the content of Fe in iron-peat (due to the coating) increased to 57 g kg^−1^. Peat sorbent had the lowest BET specific surface area 1.19 ± 0.02 m^2^ g^−1^, followed by iron-sand at 1.40 ± 0.01 m^2^ g^−1^, and iron-peat sorbent had the largest surface area of 2.16 ± 0.03 m^2^ g^−1^.

**Table 1:** Average metal (loid) concentrations in the fresh and spent sorbents ± standard deviation, mg kg^−1^ dw (*n* = 3)

Material	As	Cr	Cu	Fe	Zn
Fresh peat	21.2 ± 0.8	3.07 ± 0.12	< 0.2	12.316 ± 241	30.1 ± 3.1
Fresh iron-peat	21.1 ± 1.9	16.4 ± 1.2	28.2 ± 0.3	56.082 ± 1463	31.4 ± 2.8
Fresh iron-sand	< 0.4	20.4 ± 2.3	32.1 ± 2.2	32.527 ± 1927	44.0 ± 5.5
Spent peat	451 ± 71	4132 ± 606	4374 ± 941	12.788 ± 354	4456 ± 447
Spent iron-peat	1010 ± 183	4036 ± 453	3960 ± 778	54.956 ± 2487	3374 ± 184
Spent iron-sand	123 ± 7	232 ± 18	256 ± 15	31.069 ± 1131	122 ± 17

Table 2 presents the average EC and pH values in the eluates determined throughout the column experiment, in addition to average Eh values at the beginning and end of the experiment. At the beginning of the experiment, pH slightly above 4 was observed in peat-based eluates; acidic pH is typical for peat. The pH was above 2 in the iron-sand eluate; low pH resulted from acidic ferric-ferrous hydrosol. After 3 weeks, the pH of all eluates increased, and became 5.3 ± 0.2 (on average) which is similar to the pH of the inlet solution. EC of the eluates remained at the same level as within the inlet solution (10.9 ± 0.1 mS cm^−1^).

**Table 2:** Average EC, Eh, and pH values in the eluates ± standard deviation (*n* = 3)

Material	EC, mS cm^−1^	pH	Eh, mV	
			Start	End
Peat	10.7 ± 0.4	5.5 ± 0.3	253 ± 4	147 ± 7
Iron-peat	10.9 ± 1.5	5.1 ± 0.3	291 ± 8	163 ± 2
Iron-sand	10.9 ± 1.2	5.2 ± 0.3	521 ± 5	308 ± 8

**Fig. 1:**
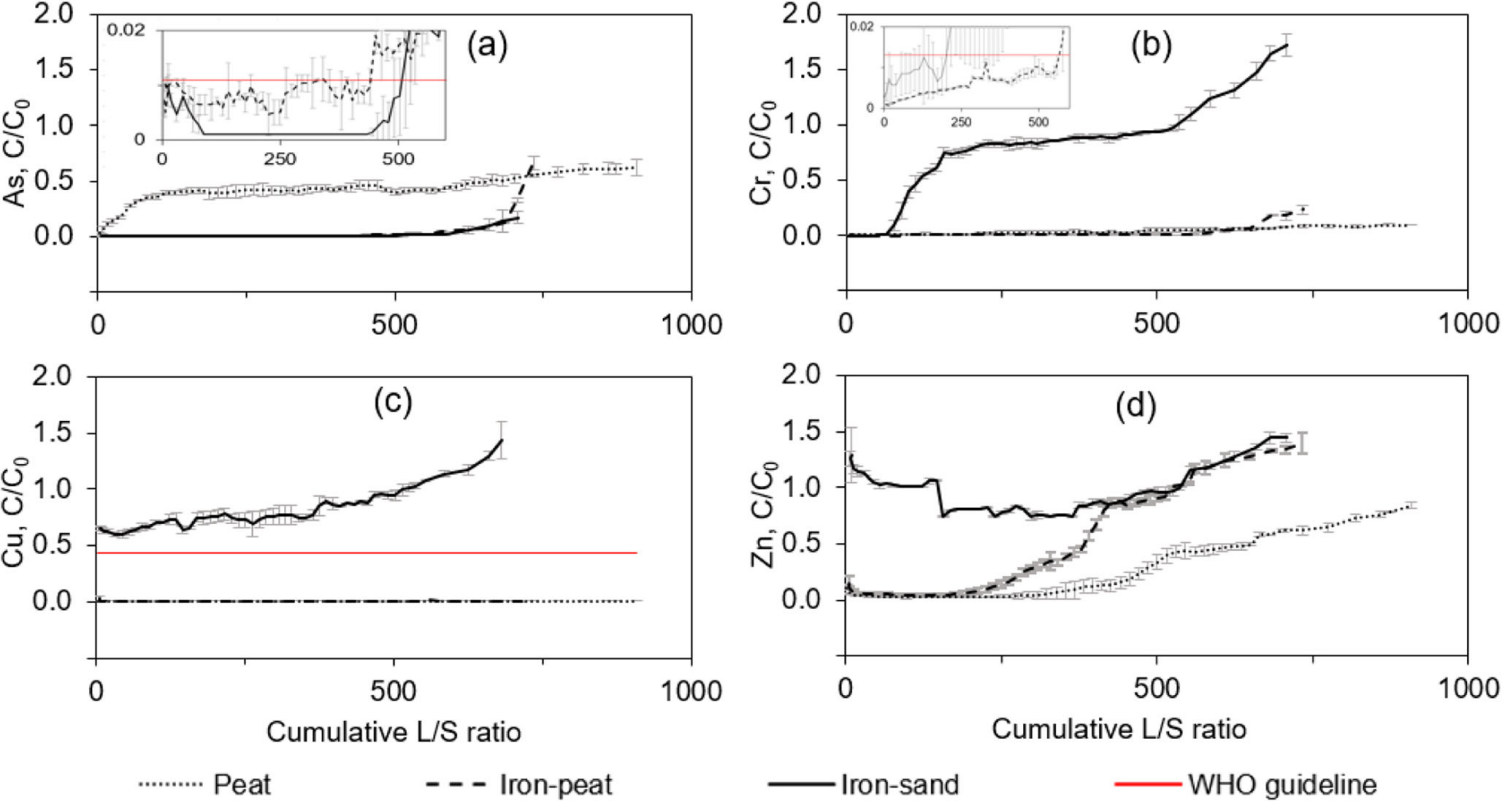
Relative metal (loid) concentrations (*C*/*C*_0_) over the cumulative L/S ratio during the column adsorption experiment. Error bars represent the standard deviation of the mean (*n* = 3). Inset figures in (a) and (b) show magnified curves at low concentrations of As and Cr. Straight vertical line denotes guideline values for drinking water proposed by WHO

Figure 1 presents relative metal (loid) concentrations (*C*/*C*_0_) over the cumulative L/S ratio. The *C*/*C*_0_ value shows the ratio between the metal (loid) concentration determined in the eluent versus the concentration in the inlet. In case the *C*/*C*_0_ ratio was higher than unity, desorption started to occur; thus, the eluent contained higher metal (loid) concentration than present in the inlet solution. Cumulative L/S ratio was chosen as a unit to express time line of the experiment. Weight of the solids (S) was fixed, whereas volume of the liquid (L) that passed through each column was increasing; therefore, the cumulative L/S ratio was increasing as well. Guideline values for the drinking water suggested by the World Health Organization (World Health Organization 2017) were used as breakthrough points, and relative *C*_WHO_/*C*_0_ values are represented by the horizontal lines in Fig. 1. The columns were filled with the same sorbent volumes, but the masses differed. A large fraction of the iron-sand was inert, and it was assumed that the sand did not affect the adsorption of metal (loid)s. To simplify a graphical presentation of the adsorption curves, the iron-sand mass was normalised, where the mass of sand was subtracted from the mass of iron-sand.

When peat was used as a sorbent, WHO drinking water guideline for As (10 μg L^−1^) was not met (Fig. 1a), and although the sorption capacity of 104 mg As g^−1^ sorbent was maintained until 4.3 BV of the contaminated solution was processed, the sorbent gradually lost its efficiency. When using iron-peat, it was possible to maintain a sorption capacity of 133 mg As g^−1^ sorbent for 220 BV before it began to lose efficiency and exceeded the WHO guideline value. Iron-sand showed a similar sorption capacity for 256 BV.

Throughout the experiment, peat showed a sorption capacity of 106 mg Cr g^−1^ sorbent (Fig. 1b), but it was too weak to meet the WHO guideline value for Cr (50 μg L^−1^). The average sorption capacity of iron-peat was 132 mg Cr g^−1^ sorbent, and 245 BV of the contaminated solution was processed below the WHO guideline value. However, when using iron-sand, it was possible to treat approximately 10 BV below the WHO guideline value, until the sorbent gradually lost its Cr adsorption efficiency.

Peat-based sorbents adsorbed Cu (Fig. 1c) with > 99% efficiency, and Cu in the eluate did not exceed the WHO guideline value (2 mg L^−1^). The average sorption capacity of peat was 109 mg Cu g^−1^ sorbent and that of iron-peat was 134 mg Cu g^−1^ sorbent, whereas iron-sand showed a Cu removal efficiency lower than 90%, with a decreasing tendency throughout the experiment.

No health-based guideline value has been proposed by WHO for Zn in drinking water. Peat and iron-peat adsorbed Zn with an efficiency of > 90% for 175 and 146 BV, respectively (Fig. 1d). The average sorption capacity of peat was 102 mg Zn g^−1^ sorbent and that of iron-peat was 118 mg Zn g^−1^, whereas iron-sand was ineffective in adsorbing Zn. Furthermore, at the beginning of the column experiment, Zn was leached out from the sand.

The Fe concentrations in eluates from peat and iron-peat were below 0.2% of total Fe, as determined in the fresh sorbents (Table 1). In the first litre of eluate from the iron-sand columns, the Fe concentration corresponded to 1.05% of the total Fe concentration in the fresh sorbent, whereas the concentration later decreased and remained below the detection limit throughout the experiment.

#### Speciation of Cr

All Cr determined in the inlet solution appeared as Cr (VI). The same was observed for the eluate samples after the first 5 days; Cr (III) was below detection limits. After 50 days of the column experiment, eluates from spent peat contained 2.28 mg L^−1^ of total Cr and Cr (VI) corresponded to 84%; eluates from iron-peat contained 1.31 mg L^−1^ of total Cr and Cr (VI) was 65%; the Cr concentration in iron-sand eluates was 4.44 mg L^−1^ and no Cr (III) was detected.

### Characterisation of spent sorbents

Loss on ignition analysis showed that spent peat and iron-peat sorbents contained 93.5 ± 0.5% and 83.3 ± 0.9% of VS, respectively, whereas the VS fraction in iron-sand was < 1%. The biochemical methane potential estimates the amount of landfill gas produced during anaerobic degradation of organic waste fraction in landfill. Over a time-span of 150 days, the control produced 23–25 mL gas g^−1^ VS, whereas spent peat and iron-peat produced significantly less gases (5–7 mL gas g^−1^ VS).

Total metal (loid) concentrations in the spent sorbents are presented in Table 1.

#### Standardised batch leaching test

After the batch leaching test, the following Eh values were measured: spent iron-sand at 174 ± 24 mV, spent peat at 188 ± 22 mV, and spent iron-peat at 210 ± 37 mV. All leachates had a slightly basic pH of 7.6 ± 0.3, and the EC values of the spent peat and iron-peat leachates were similar (116 ± 13 μS cm^−1^), whereas that of iron-sand leachates was 6.8 ± 1.3 μS cm^−1^.

Metal (loid) concentrations determined in the leachates are presented in Table 3. Percentage of the total adsorbed metal (loid) concentrations (Table 1) is also presented in Table 3. The concentrations are compared with the limit values set for waste acceptance at different landfill types according to the Council Decision 2003/33/EC.

**Table 3:** Average metal (loid) concentrations (mg kg^−1^, dw) leached from the spent sorbent during standardised batch leaching test ± standard deviation (*n* = 3), percentage [%] from adsorbed concentrations, and limit values for different landfill types (mg kg^−1^, dw)

Material	As	Cr	Cu	Fe	Zn
Peat	42.8 ± 7.9 [9.5%]	3.71 ± 0.32 [0.1%]	7.17 ± 1.15 [0.2%]	11.4 ± 3.8 [0.1%]	2.36 ± 0.47 [0.1%]
Iron-peat	9.88 ± 4.6 [1.0%]	3.65 ± 0.94 [0.1%]	6.96 ± 2.83 [0.2%]	62.3 ± 24.2 [0.1%]	1.99 ± 0.85 [0.1%]
Iron-sand	2.38 ± 1.9 [1.9%]	3.82 ± 1.13 [1.6%]	2.28 ± 4.31 [0.9%]	< 0.001 [< 0.0001%]	1.72 ± 2.01 [0.4%]
Non-hazardous waste	2	10	50	–	50
Hazardous waste	25	70	100	–	200

Nearly 10% of the adsorbed As was leached out from the spent peat sorbent, and the concentration in the leachate exceeded limit values for waste acceptable at hazardous waste landfills. However, leaching of Cr, Cu, and Zn remained < 1%, and was below the limits for non-hazardous waste landfills. Arsenic concentrations in the leachates of spent iron-peat and iron-sand sorbents defined that they could be deposited at landfills for hazardous waste. Iron-peat contained almost eight times more adsorbed As than iron-sand, but leaching from spent iron-peat was weaker. Leaching of Cr, Cu, and Zn from spent iron-peat were < 0.2% from the adsorbed content. Iron-sand leached significantly higher concentrations of Cu and Zn, and these concentrations were even more significant for Cr (1.6% from adsorbed). Leaching of Fe was minimal, especially in the case of iron-sand.

In addition to metal (loid) s, leaching of DOC was also determined. The DOC value for spent peat was 589 ± 39 mg kg^−1^, and for spent iron-peat, it was 417 ± 75 mg kg^−1^. Both values were below the limits for non-hazardous waste landfills.

#### Metal (loid) leaching at reducing conditions

The gas composition analysis identified which samples had other than landfill gases present in the bottles (Table 4). The gas composition was only measured at the end of the low redox leaching experiment. In the case of the spent peat, only one replicate out of three (peat-1) had a CH_4_ atmosphere, but O_2_ represented 5% of the total gas composition. The ratios between N_2_ and O_2_ differed in all three replicates with the spent peat, which indicates the possible occurrence of other unidentified reactions. Methane was detected in all spent iron-peat replicates, and there was no detectable O_2_. In the case of spent iron-sand, only one replicate (iron-sand-3) contained CH_4_, but O_2_ was also present at a high concentration of 11%. The ratio of N_2_ to O_2_ was similar in all bottles.

**Table 4:** Gas composition (%) in the low redox samples

Material	CO_2_	O_2_	N_2_	CH_4_	N_2_/O_2_
Peat-1	31	5	32	33	6.40
Peat-2	13	13	72	1	5.53
Peat-3	20	6	74	0	12.3
Iron-peat-1	31	1	33	35	nd^a^
Iron-peat-2	28	1	55	16	nd
Iron-peat-3	32	1	35	32	nd
Iron-sand-1	1	20	77	1	3.85
Iron-sand-2	1	21	76	2	3.61
Iron-sand-3	13	11	40	36	3.63

^a^Not detected

**Fig. 2:**
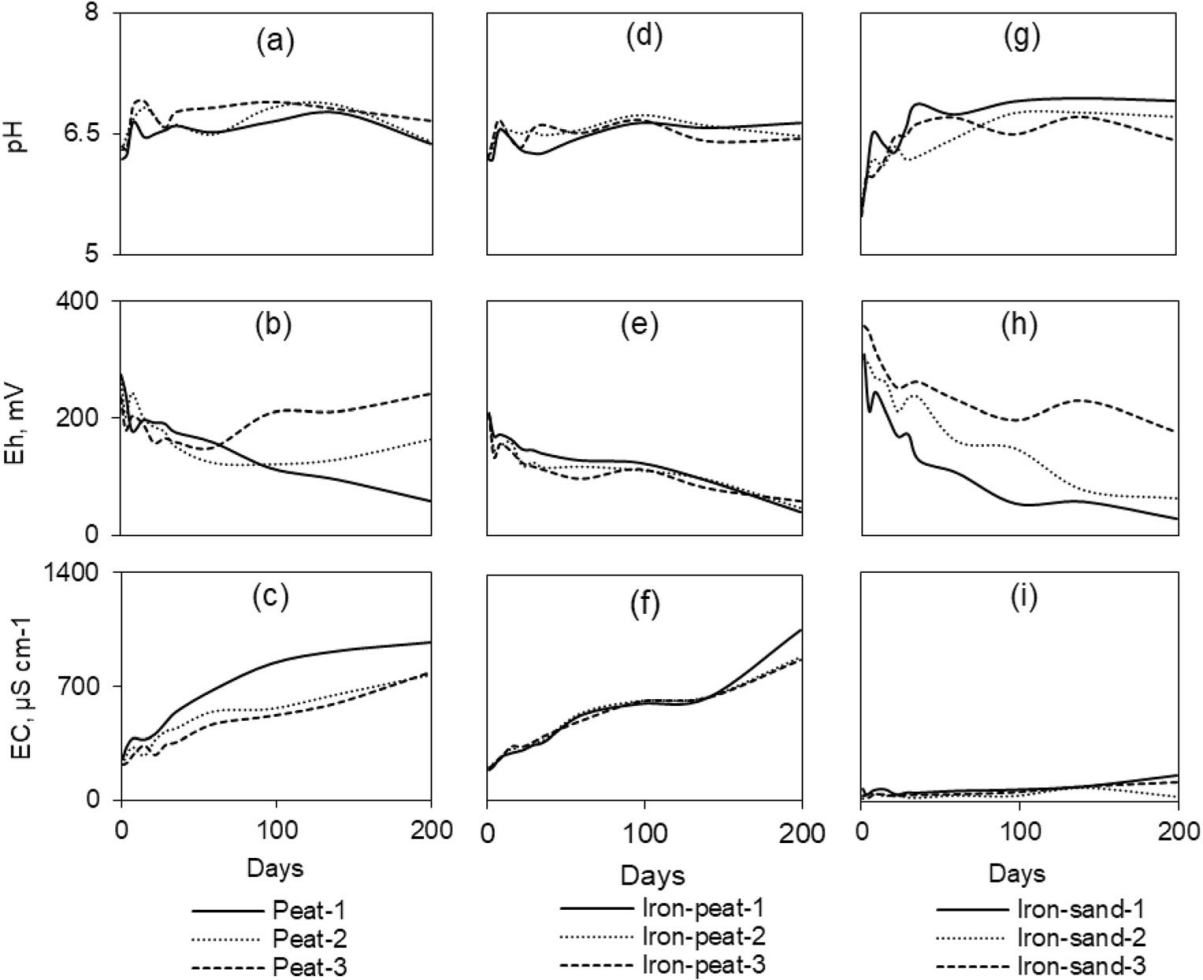
pH, Eh, and EC changes throughout the low redox experiment. A, b, c—represent triplicates with the spent peat; d, e, f—represent triplicates with the spent iron-peat; g, h, i—represent triplicates with the spent iron-sand

Determining the distinct gas composition in replicates of the same sample provided valuable insights into the different leaching behaviour of the analysed metal (loid)s. Changes in pH, Eh, and EC throughout the low redox experiment are presented in Fig. 2.

pH remained circum-neutral most of the time and showed no obvious influence from the gas composition (Fig. 2a, d, g).

Redox potential was approximately 200–270 mV in peat-based spent sorbent samples at the beginning of the experiment (Fig. 2b, e), but it then gradually dropped to 40–60 mV in replicates with a CH_4_ atmosphere. When O_2_ was present (peat-2, -3), Eh remained at a level similar to that at the beginning of the experiment, which indicated that a low redox condition had not been reached. Only iron-sand-3 maintained the CH_4_ atmosphere, whereas Eh decreased in all iron-sand replicates (Fig. 2h).

Electrical conductivity in the spent peat and iron-peat samples was approximately 190–240 μS cm^−1^ at the beginning of the experiment, and it increased continually thereafter (Fig. 2c, f). However, peat-1 showed a significantly higher EC than the other spent peat replicates. Spent iron-sand (Fig. 2i) underwent an EC increase from 47 to 105 μS cm^−1^.

The DOC content was not affected by gas composition. For spent peat, the DOC was 494 ± 4 mg kg^−1^, and for spent iron-peat, it was 483 ± 20 mg kg^−1^.

**Fig. 3:**
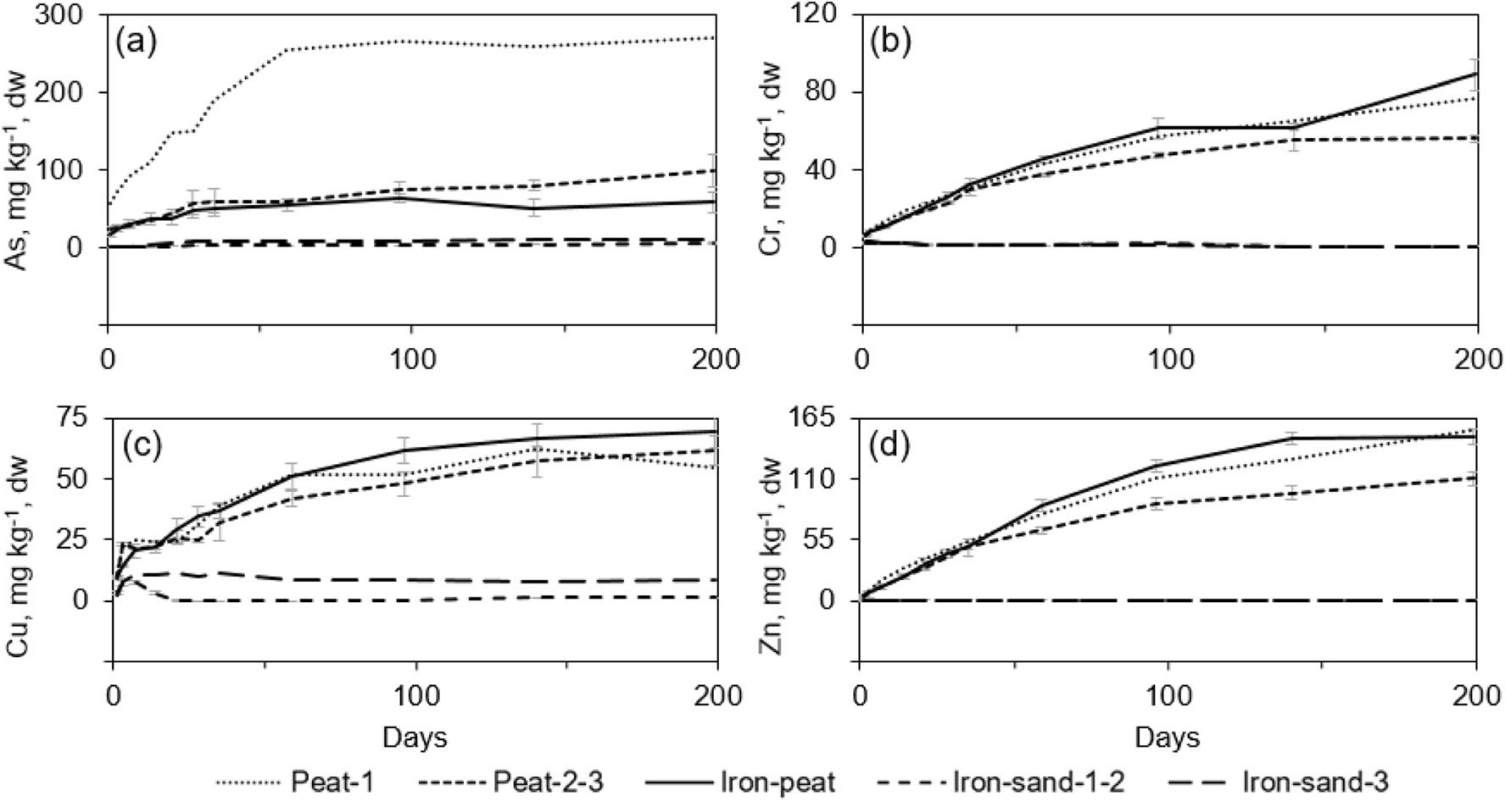
Metal (loid) leaching from spent sorbents throughout the low redox experiment. Peat-1, single replicate at reducing conditions; peat-2-3, average of two replicates at oxidising conditions; iron-peat, average of triplicate at reducing conditions; iron-sand-1-2, average of two replicates at oxidising conditions; iron-sand-3, single replicate at reducing conditions. Error bars represent the standard deviation of the mean (*n* = 2–3)

Metal (loid) concentrations in the leachates during the low redox experiment are shown in Fig. 3. The results are split depending on whether CH_4_ (or O_2_) existed in the replicates or not.

The concentrations of As (Fig. 3a) in all spent peat replicates were above the acceptance level for hazardous waste landfills (Table 3). After 200 days, 60% of the adsorbed As had been leached out from the peat-1 replicate, but a substantially lower amount (22%) of adsorbed As had been leached out from peat-2 and -3 by the end of the experiment. Leaching of As from the spent iron-peat was significantly lower than that from spent peat at reducing conditions (peat-1); although only 5.8% had been leached out at the end of the experiment, it was above the limit of disposal in hazardous waste landfills. Approximately 8% of the adsorbed As had been leached out from iron-sand-3, whereas only 3.8% of the adsorbed As had been leached at oxidising conditions (iron-sand-1, -2).

Leaching of Cr (Fig. 3b) from the peat-based spent sorbents was significantly higher at reducing conditions (peat-1 and iron-peat) in comparison with the peat-2, -3 replicates. In all cases, Cr leaching continuously increased, and by the end of the experiment, Cr concentrations of leachates were above the limit of hazardous waste landfills. However, an opposite trend was shown for Cr leaching from the spent iron-sand samples: concentrations were higher at the beginning of the experiment than at the end. However, differences in Cr leaching between samples with O_2_ (iron-sand-1, -2) and those without it (iron-sand-3) were insignificant.

The amount of Cu leached (Fig. 3c) from spent peat and iron-peat was very similar throughout all experiments, and the presence of O_2_ in the sample had no large effect on Cu leaching. However, Cu concentrations in the leachates continually increased, and after 50 days, the Cu concentration exceeded the limit of non-hazardous waste landfills. In the case of iron-sand, Cu concentrations in leachates remained at the same level throughout the experiment; however, the Cu concentration in the leachate from iron-sand-3 was up to seven times higher than those for iron-sand-1 and -2, and more than 3% of the adsorbed Cu was leached out from iron-sand at reducing conditions.

Leaching of Zn (Fig. 3d) from spent peat-based sorbents increased throughout all experiments, and after 50 days, the Zn concentration in leachates had already exceeded the limit of non-hazardous waste landfills. Reducing conditions had no effect on Zn leaching, but if based on Zn leaching only, spent iron-sand could be considered suitable for disposal at landfills for inert waste.

In the case of spent peat at reducing conditions (peat-1), 1.7% of Fe had been leached out at the end of the experiment and Fe concentrations in the leachate had gradually increased. Approximately 1.4% of Fe leached out from peat-2 and -3 during the first 50 days, and the levels remained the same level for the continued duration. Iron-peat had a manifold higher Fe content compared with the peat sorbent, but only 0.52% of Fe had leached out by the end of the experiment. However, Fe concentration in the leachates constantly increased. Iron concentrations in leachates from spent iron-sand were minimal and remained below 0.01% of the total Fe content.

#### Speciation of As

Arsenic speciation was determined in the leachates, which were obtained after completing the standardised batch leaching test and after the final low redox experiment sampling. Both MMA and DMA were found to be below the detection limit in all samples. Concentrations of As (III) and As (V) are presented in Table 5.

**Table 5:** Speciation of As in the leachates (mg kg^−1^, dw) after standardised batch leaching test and low redox experiment ± standard deviation if possible (*n* = 2–3)

Material	Concentration of species	
	As (III)	As(V)
Standardised batch leaching test, L/S = 10		
Peat	2.53 ± 0.27	6.18 ± 4.47
Iron-peat	1.57 ± 1.19	3.29 ± 2.22
Iron-sand	< 0.001	0.069 ± 0.009
Low redox experiment		
Peat-1^a^	18.6	37.3
Peat-2, -3^b^	< 0.001	61.7 ± 36.1
Iron-peat^c^	7.84 ± 0.39	18.4 ± 5.7
Iron-sand-1, -2^d^	< 0.001	0.274 ± 0.056
Iron-sand-3^e^	< 0.001	0.178

^a^Single replicate at reducing conditions

^b^Average of two replicates at oxidising conditions

^c^Average of triplicate at reducing conditions

^d^Average of two replicates at oxidising conditions

^e^Single replicate at reducing condition

At reducing conditions (peat-1), As (III) corresponded to approximately 33% of all the detected As species. However, in the presence of O_2_ (peat-2, -3), the As (III) concentration was below the detection limit and only As(V) was detected. In iron-peat samples, the amount of As (III) corresponded to approximately 30% of all determined As species.

The reducing conditions had no significant effect on As speciation in iron-sand samples. Arsenate was dominant in the leachates, and the concentration of As (III) was below the detection limits.

## Discussion

### Efficiency of sorbents

Adsorbents used to clean metal (loid) contaminated water need to be efficient and sustainable, which means that the cost of treatment should be balanced to provide both environmental and social benefits. Replacing virgin materials with residual materials when producing adsorbents increases the sustainability of the treatment. Therefore, adsorption onto low-cost particulate media, such as peat waste, is an attractive and inexpensive option for removing dissolved metals, especially divalent cations (Brown et al. 2000). At least 100 kt of peat is produced every year in Sweden (Statistics Sweden 2014), and this is mainly used in the production of electricity and energy, agriculture, horticulture, and water filtration. Peat residue is mainly obtained while milling, pelletising, and making briquettes from sod peat. However, as it usually exists as a fine powder, it is difficult to use this waste material again in the production line, as fine powders are difficult to handle and create dust problems. Nevertheless, utilising peat residuals as sorbents would decrease the volume of waste that needs management.

The heat-treated peat used in this study shows a strong potential for adsorbing divalent metal cations from contaminated solution. In the column adsorption experiment, the sorption capacity of Cu onto peat remained > 99% after processing approximately 312 BV of the contaminated solution, which indicates that the sorbent’s potential towards Cu had not been exhausted. It is considered that the favourable pH of around 5 in the metal (loid) solution is the likely reason for the efficient adsorption of Cu by peat (Ho et al. 1994). However, although the average sorption capacity of Zn onto peat was 102 mg Zn kg^−1^ sorbent, the sorbent started to become exhausted after processing 165 BV of the contaminated solution. There could be two possible explanations for this: first, the efficient adsorption of Zn on peat occurs at a pH range of 7–9 (Brown et al. 2000), and the contaminated solution used in the column adsorption experiment had a pH of about 5, which could be too low for substantial formation of carboxylate complexes with Zn (Bonnet and Cousins 1987). Second, it has been reported that competitive adsorption occurs between Cu and Zn ions for adsorption sites on peat (Duran-Jimenez et al. 2016).

The effective adsorption of Cr (VI) species onto peat is known to be around pH 1.5–3.0, and the effectiveness decreases with an increase in the pH value (Sharma and Forster 1993; Koloczek et al. 2018). In our case, more than 90% of inlet Cr was adsorbed onto peat-based sorbents at a pH of about five throughout the column adsorption experiment. From the decreasing Eh values that were determined in leachates during the column adsorption experiment (Table 2), it can be assumed that Cr (VI) was reduced to Cr (III). This observation was confirmed by the Cr speciation analysis, when a significant part of Cr in eluates towards the end of column experiment was determined as Cr (III). At a pH of above 3.7, the predominant species of Cr (III) are positively charged ions, Cr^3+^, which are mostly adsorbed onto hydroxylic groups present on the surface of peat (Koloczek et al. 5). In this respect, a pH range of 4.0–5.5 is the most favourable for efficient Cr (III) adsorption onto peat (Balan et al. 2009).

Adsorption of As onto peat was weaker than Cr, Cu, and Zn. Although, peat adsorbed nearly 50% of inlet As throughout the column adsorption experiment, a rapid loss of efficiency was observed after 3 days. The interaction between As and natural organic matter is a complex process and scientific literature provides several hypothesis about such binding mechanisms. For example, it is hypothesised that As can form covalent carbon-As bonds directly with organic matter (Buschmann et al. 2006) or through organic functional groups, such as hydroxyl groups (Warwick et al. 2005). It is widely suggested that metal cations (e.g. Fe) act as bridges between As and organic matter, forming ternary complexes (e.g. Mikutta and Kretzschmar 2011; Mak and Lo 2011). However, in Sundman (2014) and Sundman et al. (2015), it is stated that it is hard to find spectroscopic evidence for the existence of As(V)-Fe (III)-organic matter complexes even at high Fe and As concentrations.

In this study, the adsorption of As substantially increased when peat particles were coated with Fe. This increased adsorption of As onto iron-peat can be explained by the increase in the specific surface area (obtained by the BET method), which is one of the most important factors affecting sorption; the specific surface of iron-peat was by 1.8 times higher than uncoated peat. Furthermore, As has a high affinity for interacting with Fe hydroxides, and it is thus obvious that the sorbent with the highest Fe content and the largest specific surface area would have the highest As adsorption potential. However, the adsorption of Cr, Cu, and Zn onto iron-peat was slightly lower (up to 10–15%) than that of peat, and it is likely that coating peat with Fe resulted in blocking some of the organic groups. Nevertheless, the removal of cations remained within an acceptable range.

Iron-sand had a lower specific surface area than iron-peat. However, although the Fe content was also lower in iron-sand (3.25 g kg^−1^ vs 5.60 g kg^−1^), the sorption capacity was similar to that of iron-peat. Nevertheless, the lack of organic material that could provide cation exchange sites resulted in the very weak adsorption of Cr, Cu, and Zn.

In summary, Fe-coated peat sorbent showed properties that ensure a high sorption potential, and it could thus be effective for the simultaneous removal of metal (loid)s. These results agree with those of our previous study (Kasiuliene et al. 2018), where it was pointed that adjusting the pH to the target contaminant could provide even higher removal capacity.

The modification of peat with Fe compounds has gained considerable scientific attention in recent years (Ansone et al. 2012; Oliveira et al. 2015). In addition to removing As, the increased sorption of V group metalloids, such as antimony and tellurium, by Fe-modified peat sorbents has also been reported (Ansone and Klavins 2016). In contrast, the possibility of using waste-derived Fe sources for coating peat has seldom been discussed. Two million tonnes of Fe waste was produced in Sweden in 2015, and although the largest proportion is reused as raw material, approximately 20% is landfilled (Jernkontoret 2012). Therefore, using waste materials rich in Fe to coat peat (also waste-based) would not only reduce the need to use virgin materials, but also would reduce the volume of waste intended for landfill.

### Landfilling spent sorbents

One of the main questions that arises when discussing adsorption is what management strategies should be applied with respect to the spent sorbent. However, the management and regeneration of spent sorbents are seldom addressed. If the sorbent is used to adsorb oxyanion forming elements, such as As, regeneration techniques are limited and/or expensive (Verbinnen et al. 2015), and as As has a limited market, its recovery is not preferred. Thus, landfilling As-containing wastes has been the predominant management choice.

Iron-sand in this study was used as a control sorbent for iron-peat. As shown in the column adsorption experiment, both of the Fe-bearing sorbents adsorbed As to a similar extent. However, due to its large inert carrier proportion, iron-sand generates a considerable amount of spent sorbent. Because it could be disposed of at landfills for hazardous waste (based on the results from the standardised batch leaching test), and considering that the VS content in the iron-sand was < 1%, landfilling could be the most reasonable final sink.

In contrast with iron-sand, peat-based sorbents had a high VS content (up to 93%). However, landfilling waste with a high organic loading can cause the undesirable production of landfill gases, and the negative impact of landfill gas on the greenhouse effect, which has been much addressed in scientific literature (e.g. Lou and Nair 2009). For this reason, waste with more than 10% of organic content is not accepted at landfills in Europe. In certain circumstances, when type of waste does not fulfil the acceptance criteria, determined in the Council Directive 1999/31/EC, Annex II, and there is no other viable utilisation option, landfilling can be allowed if special permit is acquired. In this case, it is crucial to evaluate degradability of such waste under anaerobic conditions. In this study, BMP test, performed with spent peat and iron-peat, showed that gas production was supressed compared with the control inoculum samples. Microorganisms that are involved in anaerobic processes are sensitive not only to toxic metal (loid) s such as As, lead (Pb), or cadmium (Cd), but also to excessive doses of essential microelements, such as Cr, Cu, and Zn (Mudhoo and Kumar 2013). Furthermore, the DOC values in the leachates from peat-based spent sorbents were below the limit values for non-hazardous landfill wastes. Therefore, landfilling such waste would not contribute to the potential risks often related to highly biodegradable waste.

Based on the results of standardised batch leaching test, spent iron-peat could be disposed of in hazardous waste landfills. However, as the concentration of As in the leachates from spent peat was found to be above the limits for hazardous waste landfills, the spent sorbent needs to be pre-treated prior to landfilling. Although thermally treating As-rich waste is complicated, because volatilisation of As can start at temperatures as low as 320 °C (Helsen et al. 2003), fabric filters and electrostatic precipitators (that are present in modern waste incineration plants) can remove more than 99% of particulate matter (Jones and Harrison 2016). Given the high organic matter content present in spent peat-based sorbents, thermal treatment coupled with energy recovery could thus be a viable option.

The standardised batch leaching test showed that As mostly exceeded guideline limits in all three sorbents (Table 3). During the test, ultra-pure water was used as a leachant and aerobic conditions prevailed. However, landfills normally have anaerobic conditions. The mobility of metal (loid) s is governed not only by pH and the availability of sorption sites present in the system, but also by redox conditions, and the Eh in a landfill can drop as low as − 500 mV (Bozkurt et al. 2000). During the low redox experiment, it was possible to obtain Eh value only as low as 47.7 ± 8.9 mV (Fig. 2b, e, h). However, the mobility of redox-sensitive As was substantially affected by the decreasing Eh values. At reducing conditions, 60% of adsorbed As leached out from spent peat (peat-1), and ‘only’ 22% leached out during oxidising conditions (peat-2, -3). The several-fold increase in As leaching occurred because As(V) is reduced to a more mobile and toxic As (III) at reducing environment (Corvin et al. 1999). This is consistent with analysis conducted for As speciation (Table 5). Arsinite was undetectable during oxidising conditions, but more than 30% of total detected As was present as As (III) at reducing conditions. During the low redox experiment, leaching of As from Fe-bearing spent sorbents was significantly lower compared with from spent peat, and the same behaviour was observed during the standardised batch leaching test. These results indicate that reductive dissolution of the main As-bearing phases (Fe-Mn oxides), which is largely responsible for As being released back to the leachate (Kumpiene et al. 2009), was slower when the Fe concentration of the sorbent was higher.

The amount of As leached from the spent iron-peat during the low redox experiment exceeded the acceptable limit at landfills for hazardous waste. This outcome is different from the one obtained after the standardised batch leaching test. The low redox experiment lasted 200 days, and the prevailing reducing atmosphere may have caused decomposition of organic matter complexes (Bozkurt et al. 2000; Mikutta and Kretzschmar 2011), possibly involving As as well. Only 6% of the adsorbed As leached out from the spent iron-peat but considering that iron-peat adsorbed 1.01 g As kg^−1^, treatment of the leachate could become difficult. It would thus be necessary to pre-treat the spent iron-peat as well.

Iron-sand adsorbed slightly more than 0.12 g As kg^−1^ sorbent during the column adsorption test. However, although, nearly 8% of the adsorbed As leached out during the low redox experiment, the spent sorbent could still be considered a hazardous waste. However, as the As concentration in the leachate gradually increased over the time, increased concentrations of As would eventually be released into the leachate due to the dissolution of Fe hydroxides induced by the decreasing Eh.

The importance of microbial reduction of Fe hydroxides and thus the release of As cannot be ruled out in a full-scale landfill. Furthermore, the Eh in landfill can drop much lower than the value it reached during the low redox experiment, which would thus complicate treatment of the leachate.

The mobility of many other toxic metal (loid) s, including Cr, Cu, and Zn, is usually low under conditions generally found in mature landfills (Bozkurt et al. 2000). Therefore, based on Cr, Cu, and Zn, leaching during the standardised batch leaching test, spent peat, and iron-peat could be deposited of at inert waste landfills. However, leaching of these elements increased over prolonged contact with the leachant during the low redox experiment, and in the case of Cu and Zn, classification of landfill type shifted from the inert towards the hazardous landfill type. In addition, leaching of Cr during the low redox experiment exceeded the limit values for hazardous waste landfills, and there was also an increased leaching tendency of Cr, Cu, and Zn. Hydrolysis of organic matter, in particular hydroxylic groups, possibly occurring on the surface of peat (Koloczek et al. 5), may have caused desorption of positively charged Cr (III), Cu and Zn.

## Concluding remarks

Peat effectively adsorbed Cr, Cu, and Zn, whereas approximately 50% of inlet As was detected in the eluent. Iron-sand was effective only for adsorbing As, but Cr, Cu, and Zn were poorly adsorbed. Only iron-peat showed the simultaneous removal of all tested metal (loid)s. Therefore, a combination of two active sorbents, peat and Fe, is necessary for a single-step adsorption process. In addition, it would be beneficial to use waste-based materials to prevent the use of virgin materials in making the iron-peat sorbent and reduce the volume of waste intended for landfilling.

Leaching of As was a decisive factor in determining the landfill type for the spent sorbents. Based on the standardised batch leaching test, spent iron-peat and iron-sand sorbents could be disposed of at hazardous waste landfills. However, oxidising conditions, which prevailed during the standardised batch leaching test, could have led to an underestimation of redox-sensitive As leaching. Substantially higher amounts of As leached out from the spent sorbents at reducing atmosphere compared with the oxidising one. Furthermore, the reducing conditions caused As(V) to be reduced to the more-toxic As (III): therefore, the potential of increased amounts of As(V) and As (III) occurring in the landfill leachate is high. Decreasing Eh potential affected leaching of Cr, Cu, and Zn to a lesser extent.

## References

[CR1] Ahmad A, Richards LA, Bhattacharya P, Bhattacharya P, Polya DA, Jovanovic D (2017). Arsenic remediation of drinking water: an overview. Best practice guide on the control of arsenic in drinking water. Metals and related substances in drinking water series.

[CR2] Ansone L, Klavins M (2016). Sorption of V and VI group metalloids (As, Sb, Te) on modified peat sorbents. Open Chem.

[CR3] Ansone L, Klavins M, Robalds A, Viksna A (2012). Use of biomass for removal of arsenic compounds. Latv J Chem.

[CR4] Balan C, Bilba D, Macoveanu M (2009). Studies on chromium (III) removal from aqueous solution by sorption on *Sphagnum* moss peat. J Serb Chem Soc.

[CR5] Bhattacharya P, Mukherjee AB, Jacks G, Nordqvist S (2002). Metal contamination at a wood preservation site: characterisation and experimental studies on remediation. Sci Total Environ.

[CR6] Bonnet R, Cousins RPC (1987). On the metal content and metal ion uptake of botanically specific peat and the derived humic acids. Org Geochem.

[CR7] Bozkurt S, Moreno L, Neretnieks I (2000). Long-term processes in waste deposits. Sci Total Environ.

[CR8] Brown PA, Gill SA, Allen SJ (2000). Metal removal from wastewater using peat. Water Res.

[CR9] Buschmann J, Kappeler A, Lindauer U, Kistler D, Berg M, Sigg L (2006). Arsenite and arsenate binding to dissolved humic acids: influence of pH, type of humic acid, and aluminium. Environ Sci Technol.

[CR10] Callegari A, Ferronato N, Rada EC, Capodaglio AG, Torretta V (2018). Assessment of arsenic removal efficiency by an iron oxide-coated sand filter process. Environ Sci Pollut Res.

[CR11] Carabante I, Mouzon J, Kumpiene J, Gran M, Fredriksson A, Hedlund J (2014). Reutilization of porous sintered hematite bodies as effective adsorbents for arsenic(V) removal from water. Ind Eng Chem Res.

[CR12] Chaney RL, Hundemann PT (1979). Use of peat moss columns to remove cadmium from wastewaters. J Water Pollut Control Fed.

[CR13] Corvin DL, David A, Goldberg S (1999). Mobility of arsenic in soil from the Rocky Mountain Arsenal area. J Contam Hydrol.

[CR14] Council Decision 2003/33/EC: establishing criteria and procedures for the acceptance of waste at landfills pursuant to Article 16 of and Annex II to Directive 1999/31/EC

[CR15] Council Directive 1991/31/EC: on the landfill of waste

[CR16] Devi RR, Umlong IR, Das B, Borah K, Thakur AJ, Raul PK, Banerjee S, Singh L (2014). Removal of iron and arsenic (III) from drinking water using iron oxide-coated sand and limestone. Appl Water Sci.

[CR17] Duran-Jimenez G, Hernandez-Montoya V, Montes-Moran MA, Rangel-Mendez JR, Tovar-Gomez R (2016). Study of the adsorption-desorption of Cu^2+^, Cd^2+^ and Zn^2+^ in single and binary aqueous solutions using oxygenated carbons prepared by Microwave Technology. J Mol Liq.

[CR18] Hagner M, Romantschuk M, Penttinen OP, Egfors A, Marchand C, Augustsson A (2018). Assessing toxicity of metal contaminated soil from glassworks sites with a battery of biotests. Sci Total Environ.

[CR19] Hausladen DM, Fendorf S (2017). Hexavalent chromium generation within naturally structured soils and sediments. Environ Sci Technol.

[CR20] Helsen L, Van den Bulck E, Van Bael MK, Mullens J (2003). Arsenic release during pyrolysis of CCA treated wood waste: current state of knowledge. J Anal Appl Pyrolysis.

[CR21] Ho YS, Wase DAJ, Forster CF (1994). The adsorption of divalent copper ions from aqueous solution by Sphagnum moss peat. Process Saf Environ.

[CR22] Jambeck JR, Townsend TG, Solo-Gabriele HM (2008). Landfill disposal of CCA-treated wood with construction and demolition (C&D) debris: arsenic, chromium, and copper concentrations in leachate. Environ Sci Technol.

[CR23] Jernkontoret (2012) The steel eco-cycle. Scientific report 2004–2012. https://www.jernkontoret.se/en/. Accessed 09 January 2019

[CR24] Jones AM, Harrison RM (2016). Emission of ultrafine particles from the incineration of municipal solid waste: a review. Atmos Environ.

[CR25] Kasiuliene A, Carabante I, Bhattacharya P, Caporale AG, Adamo P, Kumpiene J (2018). Removal of metal (oid) s from contaminated water using iron-coated peat sorbent. Chemosphere.

[CR26] Koloczek H, Jaroslaw C, Zukowski W (2015) Peat and coconut fibre as biofilters for chromium adsorption from contaminated wastewaters. Environ Sci Pollut Res 23:527–534. 10.1007/s11356-015-5285-x 10.1007/s11356-015-5285-x PMC4712218 26315594

[CR27] Kumpiene J, Ragnvaldsson D, Lovgren L, Tesfalidet S, Gustavsson B, Lattstrom A, Leffler P, Maurice C (2009). Impact of water saturation level on arsenic and metal mobility in the Fe-amended soil. Chemosphere.

[CR28] Langner P, Mikutta C, Suess E, Marcus MA, Kretzschmar R (2013). Spatial distribution and speciation of arsenic in peat studied with Microfocused X-ray fluorescence spectrometry and X-ray absorption spectroscopy. Environ Sci Technol.

[CR29] Lou XF, Nair J (2009). The impact of landfilling and composting on greenhouse gas emissions—a review. Bioresour Technol.

[CR30] Lov A, Sjosted C, Larsbo M, Persson I, Gustafsson JP, Cornelis G, Kleja DB (2017). Solubility and transport of Cr (III) in a historically contaminated soil—evidence of a rapidly reacting dimeric Cr (III) organic matter complex. Chemosphere.

[CR31] Mak MS, Lo IM (2011). Influences of redox transformation, metal complexation and aggregation of fulvic acid and humic acid on Cr (VI) and As(V) removal by zero-valent iron. Chemosphere.

[CR32] Mikutta C, Kretzschmar R (2011). Spectroscopic evidence for ternary complex formation between arsenate and ferric iron complexes of humic substances. Environ Sci Technol.

[CR33] Mohan D, Pittman CU (2007). Arsenic removal from water/wastewater using adsorbents—a critical review. J Hazard Mater.

[CR34] Mondal MK, Garg R (2017). A comprehensive review on removal of arsenic using activated carbon prepared from easily available waste materials. Environ Sci Pollut Res.

[CR35] Mudhoo A, Kumar S (2013). Effects of heavy metals as stress factors on anaerobic digestion processes and biogas production from biomass. Int J Environ Sci Technol.

[CR36] Oliveira KL, Melo CA, Goveia D, Lobo FA, Hernandez MAA, Fraceto LF, Rosa AH (2015). Adsorption/desorption of arsenic by tropical peat: influence of organic matter, iron and aluminium. Environ Technol.

[CR37] Sharma DC, Forster CF (1993). Removal of hexavalent chromium using sphagnum moss peat. Water Res.

[CR38] Statistics Sweden (2014) Production, use and environmental impact: peat use in energy production has decreased five years in a row. Statistical news from Statistics Sweden and Swedish Energy Agency. https://www.scb.se/en/. Accessed 09 January 2019

[CR39] Stepanova VA, Pokrovsky OS, Viers J, Mironycheva-Tokareva NP, Kosykh NP, Vishnyakova EK (2015). Elemental composition of peat profiles in western Siberia: effect of the micro-landscape, latitude position and permafrost coverage. Appl Geochem.

[CR40] Sundman A. (2014) Interactions between Fe and organic matter and their impact on As(V) and P(V). Dissertation, Umeå University

[CR41] Sundman A, Karlsson T, Persson P (2015). Reactivity of Fe from a natural stream water towards as(V). Appl Geochem.

[CR42] Theis TL, Iyer R, Ellis SK (1992). Evaluating a new granular iron oxide for removing lead from drinking water. J Am Water Works Assoc.

[CR43] Verbinnen B, Block C, Caneghem J, Vandecasteele C (2015). Recycling of spent adsorbents for oxyanions and heavy metal ions in the production of ceramics. Waste Manag.

[CR44] Wang Y, Sun L, Han T, Si Y, Wang R (2017). Arsenite and arsenate leaching and retention on iron (hydr)oxide-coated sand column. J Soils Sediment.

[CR45] Warwick P, Inam E, Evans N (2005). Arsenic’s interaction with humic acid. Environ Chem.

[CR46] World Health Organization (2017) Guidelines for drinking-water quality: fourth edition incorporating the first addendum. Geneva 28759192

[CR47] Yurum A, Kocabas-Atakli ZO, Sezen M, Semiat R, Yurum Y (2014). Fast deposition of porous iron oxide on activated carbon by microwave heating and arsenic (V) removal from water. Chem Eng J.

